# Interactions amongst inflammation, renin-angiotensin-aldosterone and kallikrein-kinin systems: suggestive approaches for COVID-19 therapy

**DOI:** 10.1590/1678-9199-JVATITD-2020-0181

**Published:** 2021-12-06

**Authors:** Lilian Caroline Gonçalves Oliveira, Nayara Azinheira Nobrega Cruz, Bruna Ricelli, Helio Tedesco-Silva, José Osmar Medina-Pestana, Dulce Elena Casarini

**Affiliations:** 1 Nephrology Division, Department of Medicine, Universidade Federal de São Paulo (UNIFESP/EPM), São Paulo, SP, Brazil.

**Keywords:** Renin-angiotensin-aldosterone system, Kallikrein-kinin system, COVID-19, SARS-associated coronavirus, Inflammation, Coronavirus

## Abstract

Coronavirus disease 2019 (COVID-19) is a rapid-spread infectious disease caused by the SARS-CoV-2 virus, which can culminate in the renin-angiotensin-aldosterone (RAAS) and kallikrein-kinin (KKS) systems imbalance, and in serious consequences for infected patients. This scoping review of published research exploring the RAAS and KKS was undertaken in order to trace the history of the discovery of both systems and their multiple interactions, discuss some aspects of the viral-cell interaction, including inflammation and the system imbalance triggered by SARS-CoV-2 infection, and their consequent disorders. Furthermore, we correlate the effects of continued use of the RAAS blockers in chronic diseases therapies with the virulence and physiopathology of COVID-19. We also approach the RAAS and KKS-related proposed potential therapies for treatment of COVID-19. In this way, we reinforce the importance of exploring both systems and the application of their components or their blockers in the treatment of coronavirus disease.

## Background

SARS-CoV-2 is the virus responsible for the current devastating pandemic of coronavirus disease 2019 (COVID-19) [1-3]. This disease was first reported in Wuhan (China) in late 2019 and has spread worldwide with a high transmission rate that made COVID-19 be characterized as a pandemic, the first caused by a coronavirus, on March 11, 2020 [4]. Exceeding 5.0 million of deaths and 247 million of confirmed cases worldwide up to early November 2021, is inarguably that COVID-19 is the most challenging coronavirus outbreak in relation to the previous coronaviruses severe acute respiratory syndrome (SARS-CoV) and Middle East respiratory syndrome (MERS) [3,5]. 

COVID-19 was characterized as an acute respiratory disease that may turn into pneumonia with symptoms such as fever, cough and dyspnea, which can quickly progress to death. Multiple lines of evidence indicate that the COVID-19 pandemic has profound not only health effects, but also psychological, social and economic outcomes, which will probably persist for months and years to come [6,7]. Given the impact of the pandemic, we summarized the available updates on the multidisciplinary approaches for the therapeutic strategies for COVID-19 related with the renin-angiotensin-aldosterone system (RAAS) and kallikrein-kinin system (KKS) components. They are systems that may work coordinately to regulate blood pressure and electrolyte homeostasis, whose deregulation is related to numerous diseases.

## Methods

A scoping review with a thorough systematic search and screening process was developed based on the preferred reporting items for systematic reviews and meta-analyses (PRISMA) [8,9]. The search was performed in the following databases: Medline/Pubmed, SciELO and Scopus from 1949 to October 2020 publications. The selection of the papers was performed in a standardized manner by two authors independently. Possible discrepancies were analyzed by the third author and the search strategy was reviewed by all authors.

The articles were eligible for inclusion when they (a) brought the history of the discovery of the RAAS and KKS, (b) reported the components of the systems, namely protein precursors, enzymes and peptides, (c) were mainly focused in the basic principles of physiology systems, mechanisms of the diseases in which they are involved and the relevant treatments and (d) explored the relationship of both systems with COVID-19 in addition to its main characteristics and symptoms. The reviewers selected the 125 publications, discussed the results and had a consensus on the screening of the literature and consistency of the analysis. The final search results were exported into Zotero and duplicates were removed by the author.

Our work does not assess the quality of included articles, but aims to provide a preliminary picture of what has been published in the literature about the correlation between COVID-19 and the RAAS and KKS. 

## Results

### Renin-angiotensin-aldosterone system

The renin-angiotensin-aldosterone system is a cascade of hormones whose main function is to control blood pressure, through vasoconstriction in the smooth muscle of the vessels, and intravascular volume, in which there is a decrease in sodium excretion by the kidneys, mediated by aldosterone. There is a precursor, angiotensinogen, produced mainly in the liver, and in smaller amounts in several extrahepatic tissues, such as brain, heart and kidney. It is usually cleaved by renin, releasing angiotensin I (Ang I). In turn, this inactive decapeptide is processed by angiotensin I-converting enzyme (ACE), a dipeptidyl carboxypeptidase zinc-dependent releasing angiotensin II (Ang II), a peptide with important vasoconstrictive function. In addition, the ACE protease also participates in the metabolism of other peptides such as the conversion of angiotensin 1-7 (Ang 1-7) to angiotensin 1-5 (Ang 1-5), and also inactivates bradykinin (BK), a potent vasodilator of the KKS.

Ang II performs its main function by the angiotensin II receptors type 1 (AT1R) and type 2 (AT2R) activation, both of them belonging to the G protein-coupled receptor family. Most of the actions of Ang II are mediated by AT1R, such as promotion of hypertrophy, cellular proliferation and fibrosis. Both receptors are abundant in adults and are found mainly in vascular smooth muscle. However, in some pathological conditions, AT2 receptor shows an increased tissue expression and antagonizes the effects induced by AT1 receptor. The stimulation of AT2R provides vasodilation that can counterbalance the vasoconstrictor effects associated with the incitement of AT1 receptors.

Angiotensin converting enzyme 2 (ACE2), is a zinc metalloprotease that exists both as a membrane-associated form and as a secreted form, which is also known to regulate the RAAS. The name of ACE2 was given when it was discovered in 2000, because of considerable homology with ACE, 42% sequence identity and 61% sequence similarity. Moreover, ACE2 contains a single zinc-binding domain HEXXH, which is homologous to the active sites of ACE; however, it is not inhibited by ACE inhibitors [10,11].

In turn, ACE2 is able to cleave Ang I and II into angiotensin 1-9 (Ang 1-9) and Ang 1-7, respectively. Both are key elements related to cardiovascular protection, regulation of vascular tone, blood pressure, electrolyte balance and water intake [10,12], in addition to the important role in inflammation and fibrosis [13]. In this sense, ACE2 plays an important role in heart failure, in diabetic microvascular or macrovascular diseases [14] and in inflammatory lung disease [15].

Ang 1-7, which binds to the Mas receptor, exerts many positive effects on the cardiovascular system (e.g. increased endothelial function, reduced fibrosis, anti-proliferative effects on smooth muscle cells and anti-cardiac hypertrophy), as well as on other organs, such as the lungs, it exerts anti-fibrotic, anti-inflammatory and anti-apoptotic effects [16-19]. Ang 1-7/Mas axisalso is related to reduction of proinflammatory cytokines and induction of IL-10, an important anti-inflammatory cytokine [20]. 

Another axis that is modulated by the action of ACE2, which also shows beneficial biological effects is Ang 1-9/AT2R, resulting in cardioprotective effects [21,22]. Moreover, ACE2 also cleaves a single-terminal residue from several others bioactive peptides including neurotensin, dynorphin A (1-13), apelin-13, and des-Arg^9^bradykinin, here named DABK [23,24]. Thus, the imbalance in ACE2 levels is closely related to heart failure, systemic and pulmonary hypertension, myocardial infarction, diabetic cardiovascular complications and gut dysbiosis [25-27].

### Kallikrein-kinin system

The kallikrein-kinin system is a vasodilator system that also opposes the vasoconstrictor effects provided by the RAAS. The KKS is made up of kininogens, kallikreins (tissue and plasma), kinins, kininases and kinin-degrading enzymes. 

There are two forms of kininogens, high and low molecular weight kininogens (HMWK and LMWK, respectively). This inactive precursor, is synthesized primarily in the liver, then is secreted and transported in plasma, and processed by proteolytic action of kallikreins. Kallikreins are derived from inactive precursors, pre-kallikreins, they are synthesized predominantly in the liver and activated through the Hageman's factor (factor XII). Due to the factor XII role in the KKS system, its modulation is linked to formations of thrombosis and fibrinolysis [28,29].

It has been found that kallikrein exists in two different forms, plasma kallikrein, which cleaves HMWK into BK and tissue kallikrein, which processes LMWK into Lys-BK, known as kallidin. Through kallikreins, kininogen is cleaved generating kinins, biologically active peptides with vasodilatory actions, which can be processed by kininases, becoming inactive peptides.

Bradykinin, the main kinin, was discovered by a Brazilian scientist, Rocha e Silva, in the 1940s [30]. In a few years later, additional studies of Rocha e Silva, Ferreira and Vane revealed that certain peptides found in *Bothrops jararaca* snake venom potentiated the effects of BK by inhibiting its degradation especially in the lungs. Then, Ferreira discovered the bradykinin potentiating factor, BPF, it was the beginning of the inhibition of the angiotensin converting enzyme [31-33].

Based on BPF, scientists developed captopril (under the pharmaceutical name Capoten), that was the first oral angiotensin converting enzyme inhibitor and one of the most common therapies against arterial hypertension. It was considered a breakthrough because of its mechanism of action and its structure-based drug design [34]. 

BK has the ability to increase vascular permeability and causes vasodilation of arteries and veins, in addition to having mechanisms that trigger the release of others mediators, such as nitric oxide in inflamed tissues [35]. BK is also a potent pain-producing agent and its action is enhanced by prostaglandins. 

Other kinin product of the KKS is DABK, a stable and active BK metabolite originated by proteolytic action of carboxypeptidase M (CPM) and carboxypeptidase N (CPN), also known as kininase I. Increased DABK levels are responsible for increasing vascular permeability, thus promoting angioedema, and pro-inflammatory repercussions, which may be blocked by the action of ACE2, as this enzyme is able to degrade it [23].

Kinins act on target cells through receptors coupled to protein G, kinin B1 and B2 receptors (B1R and B2R). B2 receptor is the main mediator of BK and kallidin, while B1 receptor mediates the actions of DABK and des-Arg^10^ kallidin. The B2 receptor is constitutive and is expressed at a low level in healthy tissues, while B1R is widely distributed, and upregulated in tissue damage mediated by various pre-inflammatory cytokines. B1R and B2R activation induces vascular permeability [36], as well as neutrophil recruitment, and thus contributes to the inflammatory state [37].

### Interactions between RAAS and KKS

**Figure 1:**
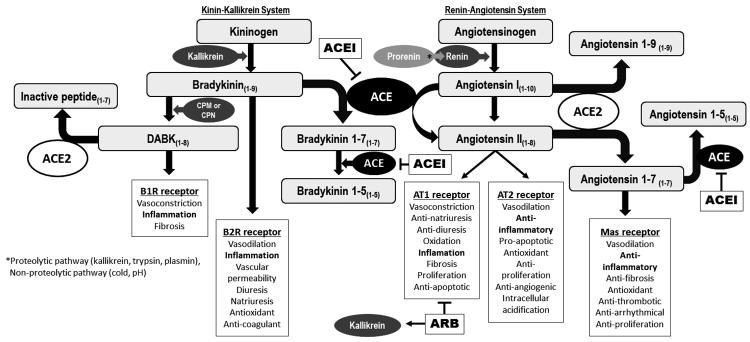
Pathways of interaction between the kallikrein-kinin and renin-angiotensin-aldosterone systems and mechanism of action of angiotensin converting enzyme (ACE) inhibitors (ACEI) and angiotensin type 1 (AT1) receptor blockers (ARB). Kininogen, the precursor of kallikrein-kinin system is cleaved by kallikrein-releasing bradykinin that acts mainly on B2R promoting the effects described in the box. Then, bradykinin is degraded by ACE or can be converted to DABK by CPM and CPN, DABK is an agonist of B1 receptor, subsequently ACE2 can inactivate DABK. In renin-angiotensin-aldosterone system, the precursor angiotensinogen is processed by renin-releasing angiotensin I, which can be cleaved by ACE to form angiotensin II that exerts its effects by binding to AT1 and AT2. Ang II is substrate for ACE2 generating angiotensin 1-7, an active peptide that exerts protective effects binding to Mas. ACEI acts by inhibiting ACE, consequently ACEIs inhibit Ang II formation and Ang 1-7 and BK degradation, these effects combined promote vasodilation. ARBs, blockers that act specifically on AT1R, inhibit Ang II binding to AT1R and its effects. Also, they possibly trigger intracellular acidification that can activate kallikrein and promote BK synthesis. B1R antagonists can block DABK-induced pro-inflammatory signaling. ACE: angiotensin-converting enzyme; ACEI: angiotensin-converting enzyme inhibitors; ACE2: angiotensin-converting enzyme 2; ARB: angiotensin II receptor blockers; B1R: kinin B1 receptor; CPM: carboxypeptidase M; CPN: carboxypeptidase N; DABK: des-Arg^9^ bradykinin.

The RAAS and KKS represent two systems with a wide range of physiological and pathophysiological actions, and play effects that are often opposite to each other. In the RAAS, the main function of ACE, is the conversion of Ang I to Ang II, but this enzyme, also known as kininase II, even is responsible for the degradation of BK, the main peptide formed by the KKS activation [38]. ACE converts BK into the thrombin-induced platelet aggregation-inhibitory peptide, bradykinin 1-5 [39,40]. In this way, ACE is the prior connection point between both systems, the RAAS and KKS, exerting important effects on kidney function modulators, as shown in Figure 1. 

Another RAAS enzyme that also plays an important role in the KKS is ACE2. Although it is unable to cleave BK, ACE2 has its metabolite as substrate, DABK. Sodhi et al. [41] provided the first evidence that DABK is a substrate of pulmonary ACE2 in vivo and the attenuation of ACE2 activity leads to decrease of DABK inactivation. Consequently, improves DABK/B1R signaling, which releases proinflammatory chemokines from airway epithelia, promotes neutrophil infiltration, and exaggerated lung inflammation and injury [41].

Concerning renin, the first enzyme responsible for cascade RAAS activation, it is obtained from prorenin processing, and it can be activated by proteolytic action, from removal its propeptide, or by non-proteolytic way, which involves conformational arrangements induced by exposure to low pH and cold [42,43]. Trypsin and plasmin, as well as tissue and plasma kallikreins can all correctly process prorenin in vitro [44-46]. Kallikrein is generated from pre-kallikrein in plasma after destruction of the natural inhibitors of contact activation, by exposure to low pH or low temperature, and probably serves as a prorenin-activating enzyme [46,47], another connection point between the RAAS and KKS systems.

Although there are no compelling evidences for in vivo prorenin activation by kallikrein, some studies indicate a correlation between kallikrein variables or levels with active plasma renin [48-50]. Lieb et al. [51] performed a meta-analysis from cohorts of European and European-American ancestry and found association between plasma renin activity and concentration with kininogen and pre-kallikrein genes. Their data add support to this concept by indicating that genetic variation in the KKS components influence interindividual variation of plasma renin activity [51].

Another factor that can modulate renin is the stimulation of B2R by BK, that induces renin synthesis and releasing, by collecting duct cells, through protein kinase C stimulation and nitric oxide release [52].In addition, PGE2 (prostaglandin E2), a product of BK stimulation, was described for releasing renin, mediated by EP2 and EP4 receptors in mouse kidneys[53],which support further the interactions between the RAAS and KKS.

Ang 1-7, which has augmented levels with increased levels of ACE2 or Ang I, can also improve BK effects. In addition to inhibiting ACE activity by binding to its active site, independently of blocking ligand hydrolysis, Ang 1-7 also is able to potentiate B2 receptor through direct or indirect interaction of its receptor with B2R after peptide stimulation [54,55]. 

Moreover, B2R forms dimers with several RAAS receptors that are important for several physiologic functions, including thrombosis risk regulation. The B2R also complexes with endothelial cell nitric oxide synthase (eNOS, NOS3), while B1R couples with cytokine‐inducible nitric oxide synthase (iNOS, NOS2) [56].

Recently, it was demonstrated that Ang II-mediated effects on neuroinflammation and oxidative stress are mediated by the stimulation of B1R, and its blockade prevents such effects in neurons in mouse neuronal cultures [57].

Although the relationship between angiotensin II receptors and the KKS has been poorly studied, it has been shown that stimulation of the AT2R by Ang II causes intracellular acidification. Since acidification is known to increase kininogenase activity, it is possible that AT2R mediates intracellular acidification and kallikrein activation, resulting in the KKS stimulation via BK releasing [58].

### Coronavirus disease 2019 (COVID-19)

Besides ACE2 had distinct roles ranging from catalytic activities with various substrates to amino acid transporter, ACE2 also plays an important role as a receptor on severe acute respiratory syndrome (SARS) coronaviruses [59-63]. SARS-CoV-2 transmission occurs through different routes (that is, fomites, air or fecal-oral route) from animal to human and human to human. COVID-19 has shown a wide variety of expression and severity of symptoms, from very mild or nonexistent symptoms to flu-like symptoms and, in more severe cases, pneumonia, severe acute respiratory syndrome and even death [64].

 In addition to the occurrence of acute injury and loss of renal function, chronic damage to the cardiovascular system is an important clinical complication of viral infection and is associated with increased rates of mortality and morbidity in these patients [65,66]. Manifestations of COVID-19 include other organs with symptoms at the digestive tract, sensory perceptions and at the central nervous system. 

A critical literature review suggests that the severity of SARS-CoV-2 infection is also associated with loss of the immune regulation between protective and altered responses due to exacerbation of the inflammatory components, which in turn inhibits the development of protective immunity to the infection [67]. Such dysregulated inflammation results in a cytokine storm that is evident in sepsis as well as in patients with severe respiratory diseases caused by coronaviruses such as SARS-CoV, MERS-CoV and SARS-CoV-2 [68,69]. In an observational study with patients with severe COVID-19 symptoms it was related exacerbated systemic inflammation and signs of T cells exhaustion [70].

The causes for these variations in disease severity are probably multifactorial, encompassing complex factors such as the expression of key components in different organs, patient health conditions, in addition to genetic factors.

The new coronavirus depends on two human proteins: ACE2, as a human receptor for virus invasion in the host cell, through interaction with viral S protein (Spike protein); and TMPRSS2, a serine protease which is responsible for the correct priming of S protein [63]. It is known that an efficient interaction of viral S protein with human ACE2 a critical step in the replication cycle and it requires a certain level of affinity between the molecules. Furthermore, the efficiency of viral infection is strongly dependent on this process.

In this sense, Ortega et al. [71] suggest that mutations in the viral S protein sequence might be favoring human to human transmission. They observed changes that triggered significant effects on SARS-CoV-2 spike/ACE2 interaction and reduced the binding energy, compared to Bat-CoV spike/ACE2 interaction [71]. Therefore, specific changes, in the nature of residues or in the type of chemical interactions occurring between ligand and receptor, may be decisive. As it generates an improvement in this affinity, or even destabilize such interaction, which might play an imperative role in the differences in susceptibility to the disease and its symptoms.

It was reported that SARS-CoV-2 is able to bind to alveolar pneumocytes, which express ACE2 on its surface [72]. However, ACE2 mRNA is also found in a much broader distribution, including upper airways, heart, blood vessels, kidneys, liver, testis, gastrointestinal tract and eyes, which opens up the possibility of this virus infecting others tissues than the lung [10,73-75]. In severe conditions of COVID-19, the presence of the viral receptor in these others tissues may explain the failure of several organs occasionally described in clinical studies. Therefore, it is not surprising that the initial reports have suggested that hypertension, diabetes, cerebrovascular and coronary heart diseases are the most frequent comorbidities in COVID-19 [76,77].

The difference in responses to SARS-CoV-2 infection between different individuals and countries can also be explained by the decreased immune response in the elderly, the presence of comorbidities or smoking habits [78]. However, severe cases of COVID-19 have been observed in young people, apparently without risk factors, as well. It indicates that most of the factors that explain the severity of the disease are still unknown.

### The role of RAAS and KKS in COVID-19

The correlation between the RAAS and KKS with COVID-19 pathogenesis is suggested by several clinical features and symptoms observed in patients, given the close interconnection between both systems. While the RAAS controls vasoconstriction and vasodilation, the KKS regulates vasodilation and vascular permeability, which are also important in COVID-19.

It is known that the virus survival strategy is to elude and suppress host innate immune defenses through gene deactivation or inhibition [59,79]. In line, in coronaviruses as SARS-CoV, studies were observed a viral nonstructural protein (nsp1) binding to ribosomes and inhibiting host gene translation, a marked downregulation of ACE2 expression and inducing of ACE2 shedding from the cell surface [80-82].

Due to the important pathophysiological role of ACE2, Samavati and Uhal [83] state that the binding of the viral spike to ACE2 triggers a potential effect on the loss of the protective effect of the ACE2/ Ang 1-7/ Mas pathway on alveolar epithelial cells and other organs, in addition to causing increased levels of Ang II, which can amplify the systemic deleterious effects of the RAAS in the patients [83]. Although ACE2 allows viral entry at the epithelial surface, the ACE2/Ang 1-7/Mas axis can represent a potential target for therapeutic intervention, due to its role in protection in acute lung injury.

Compared to women, men with COVID-19 have more severe disease and higher mortality [84,85], it can be explained by several risk factors that are more frequent in men. Such as higher rates of preexisting comorbidities associated with COVID-19, as ischemic heart disease, hypertension, diabetes, chronic renal disease and cancer within 5 years, higher risk behaviors, as smoking and alcohol use, social isolation and certain occupational exposures; and lower innate immune response [86].Regarding the difference in ACE2 levels between genders, the ACE2 gene is located on the X chromosome and the testis have much higher levels of ACE2 than the ovaries, which also suggests that women might have higher ACE2 levels and thus be protected against more severe disease compared to men [87,88]. 

In vivo, the enzyme that mediates the shedding of ACE2 ectodomain is ADAM-17 (A- disintegrin and metalloproteinase 17), also known as TACE (TNF-(-converting enzyme) due to its role to driven tumor necrosis factor-( (TNF-() extracellular domain shedding and activation, a cytokine implicated in chronic inflammation [89,90]. In addition, ADAM 17 is related to the processing of other cytokines and receptors, among which many are correlated with the initiation and exacerbation of inflammatory process [91].

After the binding of SARS-CoV-2 to ACE2, the complex is internalized by endocytosis and ACE2 shedding is induced, consequently, the diminished ACE2 availability impairs directly the protective roles of ACE2/Ang 1-7/Mas and Ang 1-9/AT2R axes. In turn, the increase in Ang II levels activates the AT1 receptor, the overactivation of this signaling induces deleterious actions as progression of cardiovascular diseases, end-organs injury, cell growth, vascular contraction, fibrosis, inflammatory responses and salt and water retention, which undoubtedly, on its own, impairs the health of the infected patient [92]. 

Furthermore, other consequence of increased signaling via AT1R, is the activation of ADAM-17, which triggers the TNF-( releasing into extracellular region. In addition to the systemic cytokines, released due to SARS-CoV-2 infection, it can lead cytokine storm, besides to result in loss of ACE2 at the membrane due to its shedding function, leading a RAAS positive feedback cycle [92-94].

Regarding the KKS, Nicolau et al. [95] was the first group that linked bradykinin to COVID-19 context, correlating it with Sérgio Ferreira's contributions from basic science to clinical ambit and this system. They hypothesized that targeting the KKS may be beneficial in SARS-CoV-2 infection, especially on early stages [95].

 The interaction of kinins to their respective receptors will increase the activation of eNOS and iNOS. It ensues nitric oxide and prostacyclin (PGI2) releasing along with pro-inflammatory cytokines/chemokines responsible for acute inflammation, which cause vasodilation, pain, cell proliferation and fibrosis [96,97], typical symptoms of COVID-19.

A common feature for many patients that get severe COVID-19 is serious lung damage caused by an overly vigorous immune response. It is characterized by the production of numerous inflammatory cytokines, the consequent cytokine storm. Ferreira et al. [98] demonstrated that BK production is an important step in the activation of a cascade of cytokines that participate in the inflammatory hyperalgesia [98]. 

COVID-19 patients can present with pulmonary edema early as symptom of the viral infection. Van de Veerdonk et al. [99,100] proposed that this disorder is caused by a local vascular problem due to activation of KKS receptors in lung endothelial cells. Since the coronavirus blocks ACE2 proteolytic activity for cell infection, the enzyme is unable to inactivate DABK, the potent ligand of B1R. Under this condition the lung environment is prone for a kinin-dependent local vascular leakage leading to angioedema via B1R and eventually B2R. Since this disorder is resistant to corticosteroids or adrenaline, this is an important feature of COVID-19 [99,100].

KKS appears to be involved in vascular leakage and inflammatory response observed during different viral infections [101]. Another symptom that has been highlighted in patients with COVID-19 and seems to be involved with this system is the formation of microthrombi, which can trigger thrombosis [102]. Since kallikrein, additionally, causes imbalance of coagulation system by activating factor XII and plasmin, the two mechanisms contribute to the formation of intravascular microthrombi, observed mainly in the lung tissue [95].

### RAAS blockers and related COVID-19 therapies

RAAS blockers, as ACE inhibitors (ACEI) and angiotensin II receptor blockers (ARBs), are worldwide used for effectively reducing systemic vascular resistance in patients with hypertension, heart failure and chronic renal disease. There are animal models studies that demonstrated a substantial increase of ACE2 expression under ACE inhibitors or ARBs administration, which represents a potential mechanistic link between SARS-CoV-2 infection and these medications [103-105].

ACEI group (such as enalapril, ramipril, captopril, and lisinopril) acts by inhibiting ACE, making it unable to convert Ang I into Ang II, thus blocking vasoconstrictor properties, the activation of aldosterone and sodium reabsorption attributed to Ang II of the RAAS. In addition, the ACEI inhibits the conversion of active kinins (such as bradykinin and kallidin) in inactive peptides, promoting the vasodilating effect of the KKS cascade.

Evidences suggest that ACEI can also abolish the desensitization of the B2R or delay its sequestration, which also potentiates BK action [106]. Another effect of ACEI on KKS receptors was demonstrated by Ignjatovic et al. [107], they found that enalaprilat activates B1R directly in the absence of ACE. This inhibitor activates at the zinc-binding consensus sequence HEXXH in B1 receptor, which is present also in ACE but not in B2R [107].

While the ARB group (such as losartan, candesartan, valsartan, irbesartan, and telmisartan) blocks the AT1R. This blockade leads to increased Ang II levels, which stimulates the non-blocked angiotensin II receptor, AT2R, and triggers intracellular acidification by inhibiting the amiloride-sensitive Na^+^/H^+^ exchanger. 

Consequently, it is possible that this condition activates kallikrein, resulting in augmented BK production and endothelial B2R stimulation through a paracrine mechanism, activating the NO/cGMP system and causing vasodilation [55,58]. Therefore, in addition to blocking the classical harmful effects of Ang II through AT1R signaling, therapies that use ARBs also promote the beneficial effects of the KKS, even indirectly by kallikrein activation.

The RAAS blockers have been extensively used to treat cardiovascular disorders, reducing mortality and morbidity. Thus, researches on SARS-CoV-2 pathogenesis have been focused on discussions about these compounds, and how they could fit pathophysiological processes of COVID-19, since these pathways were substantially studied in SARS-CoV [76,108]. Due to the increased ACE2 expression caused by treatment with these popularly used classes of drugs in some experimental models and high number of infected hypertensive patients, questions such as whether continued use of RAAS blockers could increase virulence and severity of symptoms or whether such treatment should be stopped, have been frequently asked.

The relationship between hypertension and COVID-19 mortality exists, but it is known that older ages strongly correlate with hypertension and have been associated with higher mortality rates from COVID-19 [85,109]. Thus, new analyses need to be done aiming an adjustment with age-stratified data for the hypertension and mortality association, to verify if this is a reliable finding [110].

Evidences also supports the possibility that ACEIs and/or ARBs could reduce the severity of COVID-19 infection, since ACEI and/or ARB treatment could diminish effects of Ang II and increase Ang 1-7 effects, leading to attenuated inflammation and fibrosis [111]. Some studies, with animal models, support the idea that RAAS blockers use could be protective in conditions of viral pneumonia, including coronavirus infection [112,113]. In addition, a recent retrospective observational study from Wuhan linked the ACEIs/ARBs treatment of hypertensive subjects affected by SARS-CoV-2 with lower all-cause mortality [114].

Interrupting ACE inhibitors and ARBs treatments in asymptomatic and stable patients with heart failure, kidney disease, diabetes or hypertension will disrupt clinical care and strongly require additional medical visits [111]. However, this decision is not feasible in this pandemic context, given the recommendations of social isolation and overcrowded hospitals.

Taking into account chronic patients using ACEI/ARBs, who were infected by SARS-CoV-2, the medical community substantially recommends they should not discontinue the treatment, neither temporarily [111,115]. Unless there exist evidence-based indication and robust data to discontinue these important life-saving medications.

Functionally, there are two forms of ACE2, the full-length ACE2 that contains a structural transmembrane domain, which anchors its extracellular domain to the plasma membrane and soluble Angiotensin Converting Enzyme 2 (sACE2) that lacks the membrane anchor and circulates in small amounts in the blood [116], other RAAS-related therapy which has been approached for COVID-19 treatment. Increasing ACE2 activity in systemic, not tissue, by human recombinant sACE2 administration may provide a new therapeutic target in states of Ang II-dependent hypertension by enhancing Ang II degradation and increasing Ang 1-7 levels [116].

In vitro study showed that SARS-CoV replication was blocked by a soluble form of ACE2 in monkey kidney cell line, Vero-E6 [59]. SARS-CoV-2 have limited potential to escape sACE2-mediated neutralization, since its binding with the virus blocks S protein and prevents its interaction with full length ACE2. Consequently, coronavirus attachment and internalization in the host cells is impaired [108], an outcome that attenuates virulence [117].

Based on these findings, Batlle et al. [108] proposed that sACE2 may act as a competitive interceptor of SARS-CoV-2 and other coronaviruses by preventing binding of the viral particle to the surface-bound, full-length ACE2. They suggest that the use of sACE2 as a potential approach for coronavirus infection therapy should be urgently tested [108].

As already described, ACE2 enzymatic functions protect against organ injury by cleavage and disposal of Ang II and the formation of Ang 1-7, as well as cleaving DABK, which is a proinflammatory peptide. In line, there are pharmacokinetic studies regarding human recombinant sACE2 in healthy volunteers and clinical trials have been developed as treatment for acute respiratory distress syndrome [118-120]. Very recent cell-based assays using engineering human sACE2 have been developed to optimize its binding to SARS-CoV-2 [117]. Using human organoids models, Monteil et al. [121] demonstrated that SARS-CoV-2 can directly infect human blood vessel and kidney organoids, and human sACE2 can inhibit these viral infections [121]. There are no studies in vivo ensuring efficacy of sACE2 human therapy yet. Furthermore, it is worth mentioning that there is a concern that blood pressure could fall excessively due to systemic inactivation of Ang II by sACE2 administration. On the other hand, increased levels of intrinsic sACE2 in COVID-19 patients can represent an additional risk, as the ACE2 anchored in cell membrane would lacks its N-domain after shedding, thus ACE2 local effects mediated by its catalytic action may decrease.

In late phases of COVID-19 development, when antiviral treatments are not so effective and ACE2 is markedly downregulated, the administration of ACE2 activator agents becomes a daring therapeutic proposal. Diminazene aceturate is an old antiparasitic that has been substantially studied due to its ACE2 activators properties, which restore protective RAAS and KKS axes [122]. 

As this compound has anti-inflammatory and tissue protectant profile, in addition to be an FDA-approved drug [122,123], Nicolau et al. [124] hypothesized the use of diminazene aceturate as potential therapeutic strategy for late stage mainly in pulmonary complications provoked by SARS-CoV-2 infection. It could improve clinical outcomes by reduction of proinflammatory cytokines and augmenting surfactant proteins ACE2-dependent, consequent effects of ACE2 activation [124].

**Figure 2:**
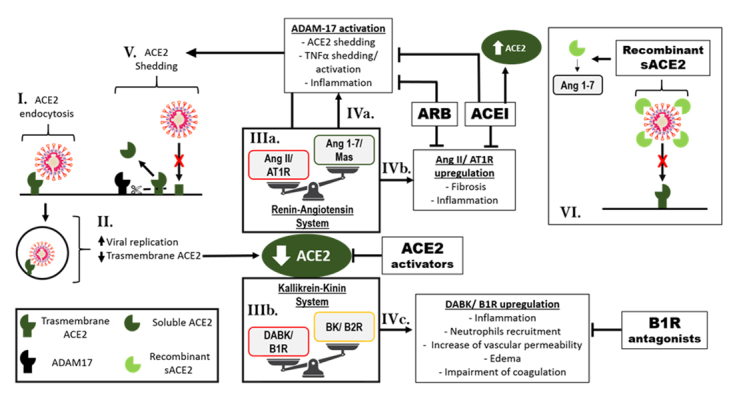
Schematic of COVID-19 outcomes on the renin-angiotensin-aldosterone and kallikrein-kinin systems, and proposed therapies with RAAS blockers, B1R and recombinant sACE2. **(I)** SARS-CoV-2/ACE2 complex is internalized by endocytosis, **(II)** triggering viral replication and reduction of transmembrane ACE2, which provokes **(IIIa)** the imbalance of RAAS and KKS, with **(IIIb)** Ang II/AT1R and DABK/B1R pathway activation, respectively. **(IVa)** Ang II/AT1R upregulation induces ADAM-17 activation, which is responsible for **(V)** ACE2 shedding that can contribute to depletion of ACE2 local effects, in addition **(IVb)** Ang II/AT1R promotes inflammatory and fibrotic processes. **(IVc)** DABK/B1R upregulation also triggers pro-inflammatory cascades. It is pointed out the actions of RAAS blockers (ACEI and ARB), ACE2 activators and B1R antagonists, counterbalancing the deleterious effects of downregulation of ACE2 in SARS-CoV-2 infection. In detail, **(VI)** the effect of sACE2 recombinant as therapy, that act as a competitive interceptor of SARS-CoV-2 by preventing binding of coronavirus to the surface-bound, besides providing increased Ang 1-7 circulating levels. ACEI: angiotensin-converting enzyme inhibitors; ACE2: angiotensin-converting enzyme 2; ADAM-17: A-disintegrin and metalloproteinase 17; Ang 1-7: angiotensin 1-7; Ang II: angiotensin II; AT1R: angiotensin II receptor type 1; ARB: angiotensin II receptor blockers; BK: bradykinin; B1R: kinin B1 receptor; B2R: kinin B2 receptor; DABK, des-Arg^9^ bradykinin; KKS: kallikrein-kinin system; RAAS: renin-angiotensin-aldosterone system; sACE2: soluble angiotensin converting enzyme 2.

Taken together, the protective effects of RAAS blockers on the heart and blood vessels are at least partly mediated by the direct or indirect KKS activation, and reduction of Ang II/AT1R signaling, due to decreased Ang II production or AT1R blockade, increase of Ang l levels and consequently augmented Ang 1-7 by the action of Neprilisin. Or in the case of sACE2 therapy and ACE2 activators, there is a counterbalancing of the deleterious effects caused by downregulation of ACE2 in SARS-CoV-2 infection, as shown in Figure 2. In this way, it occurs a direct increased formation of protective angiotensin, Ang 1-7 from Ang II and Ang 1-9 from Ang I, in addition to reducing DABK levels and its inflammatory outcomes.

Potential therapeutic strategies related with the RAAS components also comprise the prevention of S protein SARS-CoV-2/ACE2 interaction by viral receptor-binding domain blockade. Which may include the use of ACE2-derived peptides, small molecule inhibitors, ACE2 antibody or single chain antibody fragment against ACE2[92].

The enhancement of DABK/B1R signaling, caused by reduced ACE2 levels after coronavirus infection, triggers consequential events as fluid extravasation, leukocyte recruitment to the lung and may increase the risk of capillary permeability, acute respiratory distress syndrome and multiple organ failure [41]. Administration of B1R antagonists in experimental models of sepsis shown prevented hemodynamic derangement and attenuates the risk of multi-organ failure [125].

Regarding COVID-19 patients that present pulmonary edema early in disease, this condition added to enhancement of local immune cell influx and proinflammatory cytokines leading to damage, has been resulting in a very high number of intensive care unit admissions. It was hypothesized that blocking the B2R and inhibiting plasma kallikrein activity might have an ameliorating effect on early disorders caused by COVID-19 and might prevent acute respiratory distress syndrome [99,100], besides being able to collaborate with indirectly response to anti-inflammatory agents. Thus, there are indications that the KKS receptors antagonists can also be an option for symptoms COVID-19 treatment.

## Conclusion

SARS-CoV-2 infection is intrinsically related to the RAAS, as the viral internalization apparatus is driven by ACE2, and indirectly linked to the KKS, due to the action of this enzyme on the degradation of DABK. Imbalance in the RAAS and KKS (caused primarily by loss of ACE2 activity in patients with COVID-19 are contributing factors to consequent deregulation of blood pressure), loss of protective effects, organ damage and exacerbated tissue and systemic inflammation, among others, are key factors that may explain the severity of the disease. These conditions make it tricky for the organism to react against the pathogenesis considering that, per se, it weakens the health conditions of patients.

Even with the proven increased ACE2 levels in continuous treatment with RAAS blockers, in view of its protective role, scientific communities strongly suggest a rationale for continuing this therapy in patients with COVID-19 infection. In addition, since it is responsible for metabolizing Ang II into Ang 1-7, a dominant mechanism for negative regulation on the RAAS, and for inactivating DABK, an inductor of pro-inflammatory repercussions of the KKS, ACE2 has relevant properties that have to be further explored as tools for the treatment of COVID-19 patients. Taken together, there are gaps in knowledge that highlight the need for new studies to design more effective therapeutic and prophylactic strategies.

### Abbreviations

ACE: angiotensin I-converting enzyme; ACEI: ACE inhibitor; ADAM-17: A-disintegrin and metalloproteinase 17; Ang 1-5: angiotensin 1-5; Ang 1-7: angiotensin 1-7; Ang 1-9: angiotensin 1-9; Ang I: angiotensin I; Ang II: angiotensin II; ARB: angiotensin II receptor blocker; AT1R: angiotensin II receptor type 1; AT2R: angiotensin II receptor type 2; B1R: kinin B1 receptor; B2R: kinin B2 receptor; BK: bradykinin; BPF: bradykinin potentiating factor; COVID-19: coronavirus disease 2019; DABK: des-Arg^9^bradykinin; eNOS: endothelial nitric oxide synthase; HMWK: high molecular weight kininogen; iNOS: inducible nitric oxide synthase; KKS: kallikrein-kinin system; LMWK: low molecular weight kininogen; Lys-BK: lysine-bradykinin; MERS: Middle East respiratory syndrome; PGE2: prostaglandin E2; RAAS: renin-angiotensin-aldosterone system; S protein: spike protein; sACE2: soluble angiotensin converting enzyme 2; SARS: severe acute respiratory syndrome; TACE: TNF-(-converting enzyme; TNF-(: tumor necrosis factor-(.
